# A 4-Year Follow-Up Study of Attention-Deficit Hyperactivity Symptoms, Comorbidities, and Psychostimulant Use in a Brazilian Sample of Children and Adolescents with Attention-Deficit/Hyperactivity Disorder

**DOI:** 10.3389/fpsyt.2015.00135

**Published:** 2015-10-02

**Authors:** Sonia M. M. Palma, Ana Carolina M. P. Natale, Helena M. Calil

**Affiliations:** ^1^Department of Psychobiology, Universidade Federal de São Paulo (UNIFESP), São Paulo, Brazil; ^2^Universidade Mackenzie, São Paulo, Brazil

**Keywords:** ADHD, comorbidity, follow-up, remission, school dropout, psychostimulant

## Abstract

The aim of this study was to evaluate symptom persistence in attention-deficit/hyperactivity disorder (ADHD), the development of comorbidities, and psychostimulant usage patterns. Follow-up studies were conducted in 37 patients with ADHD and 22 healthy controls, aged 10 and 18, 4 years after their first assessment. The ADHD was rated as persistent if participants met all DSM-IV criteria for syndromic or sub-threshold persistence, or had functional impairments (functional persistence). Of the 37 ADHD patients we reevaluated, 75% had persistent symptoms, and psychiatric comorbidities with additional functional impairments and academic problems were more common than in controls. These follow-up findings show a high comorbidity associated with ADHD and support the importance of evaluation and treatment for ADHD and comorbidities throughout life.

## Introduction

Attention-deficit/hyperactivity disorder (ADHD) is a childhood-onset neurodevelopmental condition characterized by age-inappropriate levels of inattention, hyperactivity, and impulsivity (1), and it is the most common of the neurodevelopmental disorders (2). It is multifactorial and clinically heterogeneous (3), with a worldwide prevalence rate of 5.3% (4) and a prevalence rate of 5–10% in school-aged children (5, 6).

Although ADHD is a neurodevelopmental disorder, hyperactivity–impulsivity symptoms declined with increasing age, but inattention symptoms did not (7–9), and many patients maintain symptoms into adolescence (75%) and adulthood (66%) (10–12).

Moreover, ADHD is frequently associated with psychiatric comorbidities, such as anxiety disorders (30–40%), (13, 14), and conduct disorder (CD)/oppositional defiance disorder (ODD) (30–50%), (10). Psychiatric comorbidities might influence the clinical presentation of ADHD and may hinder the differential diagnosis of disorders. Diagnoses of ADHD can be easily confused if symptoms of impulsivity are decreased with anxiety or if symptoms of inattention are seen as secondary to comorbid psychiatric conditions (15).

Thus, the persistence of ADHD symptoms is a risk factor for a number of adverse outcomes in adolescence and adulthood, especially when there are comorbidities. It is associated with continuing social, educational, occupational, interpersonal difficulties, substance use disorders, driving difficulties and accidents, and delinquency problems (1, 8, 16–18). Thus, the associations between the high prevalence, the persistence of symptoms, and the presence of comorbidities make the study of ADHD very interesting, important, and challenging.

There have been several follow-up studies of children documenting the persistence of ADHD into adolescence and adulthood (7, 12, 19–34). However, almost all of these studies were conducted in clinical samples in North America. Culture can affect different aspects of development, such as language development, behavioral expectations, parental tolerance for non-compliant behaviors, emotional regulation, binding styles, identity formation, motivation, and other aspects of parenting and early childhood education (35). Therefore, the diagnosis of ADHD and its evolution may be affected by cultural factors. Thus, it is difficult to generalize these results to other countries. In Brazil, there is a lack of follow-up of patients with ADHD diagnoses. A brief survey of the literature found only two follow-up studies in our country, by the same group of researchers (25, 36).

Thus, a prospective longitudinal follow-up study was designed to (a) investigate ADHD symptom persistence and full remission over these 4 years and to (b) track comorbidities and psychostimulant use.

## Materials and Methods

The project was approved by the Research Ethics Committee of UNIFESP-HSP (protocol # 1616/06). All patients, healthy controls, and their families were informed of the procedures involved. Those who agreed to participate signed an informed consent form.

### Baseline

The baseline sample was selected from January 2007 to December 2007. This sample included children and adolescents with ADHD, visiting the Interdisciplinary Neuropsychological Child Care Center (NANI), Department of Psychobiology, UNIFESP. NANI is an outpatient facility specialized in interdisciplinary research on diagnosing neurodevelopmental disorders, delivering care, researching and teaching, specialized in ADHD. The sample at baseline consisted of 38 patients (31 boys, 7 girls), diagnosed with ADHD at a mean age of 8.7 years (SD = 2.3). The healthy subjects were matched to the ADHD patients for gender, age, level of education, and type of school attended (i.e., public or private). Their mean age was 8.8 years (SD = 1.8).

The inclusion criteria were as follows: (1) a full diagnosis of ADHD according to DSM-IV, (2) no comorbid mental disorders, (3) no history of psychostimulant medication use or any other medication that might interfere with cortisol secretion, (4) no history of a serious medical illness, and (5) an IQ score higher than 75 points. These strict criteria were responsible for the small number of participants.

### Follow-up

At follow-up, 4 years later, 37 patients (30 boys and 7 girls) and 22 healthy subjects (15 boys and 7 girls) were included. Their ages ranged from 10 to 18. They were contacted by mail, e-mail, telephone, or through their school. In the ADHD group, there was a loss of one patient for failure to locate. The control group lost 16 children and adolescents, 8 for failure to locate and 8 because the family refused to participate in the research.

### Diagnostic evaluation

#### Baseline

The diagnosis of ADHD was based on the application of a Brazilian version of the SNAP-IV (Swanson, Nolan e Pelham – version IV) and the Child Behavior Checklist (CBCL). The SNAP-IV is a questionnaire consisting of symptoms from the Diagnostic and Statistical Manual – Fourth Edition – DSM-IV (37) that has already been validated in Brazil (38). The CBCL is a questionnaire that assesses social competence and behavioral problems from ages 4 to 18 years, from information provided by the parents, previously validated in Brazil (39). Both were answered by parents or guardians. A social evaluation was conducted with the Brazil Criteria of Economic Classification, a self-reported questionnaire – the ABEP (40). All assessments were conducted by two board-certified medical doctors (one psychiatrist and one pediatric neurologist) with many years of experience (over 25 years) in clinical evaluation and research. The children underwent a battery of neuropsychological tests. At the end of this evaluation, their cases were discussed by the service team and the diagnoses established.

#### Follow-Up

Behavioral and comorbidity symptoms were reassessed using the CBCL questionnaire. The CBCL has been widely used for screening comorbidity disorders in samples of patients with ADHD (41–43).

Symptom persistence was defined based on the concepts proposed by Biederman et al. (17, 29): (a) subjects meeting full DSM-IV criteria for ADHD (“Syndromic Persistence”); (b) subjects meeting sub-threshold DSM-IV criteria [more than half of the symptoms required for a full diagnosis (“Symptomatic Persistence”)]; (c) subjects not meeting criteria (a) or (b) but functionally impaired as assessed by the global assessment of functioning (GAF) (37) and displaying a score ≤60 (“Functional Persistence”) in the month prior to the subject’s 4-year follow-up assessment. The social reevaluation used the ABEP.

#### Statistical Analysis

Statistical Package for the Social Sciences (SPSS) version 20.0 was used for all statistical analyses. The data were analyzed using descriptive statistical procedures for analyzing and testing hypotheses according to the nature of the variables (quantitative or qualitative) and the sample type (independent or paired). The normality analysis in the samples distribution was performed using Kolmorov–Smirnov test. For samples with normal distribution, parametric tests were adopted. The analysis of quantitative variables, involving the comparison of independent samples, used one-way ANOVA and Student’s *t*-tests. The analysis of qualitative variables in independent samples used the chi-squared test. For data collected longitudinally (including the number of symptoms from the DSM-IV), involving paired samples, the Split-Plot ANOVA for repeated measures, and Student’s *t*-test for paired samples were used. For all tests, a significance level of 5% was established.

## Results

### Demographic results

Thirty-seven out of the 38 previous ADHD-diagnosed patients were evaluated in the follow-up study. The mean age of the adolescents was 13.1 (SD = 2.5) for the remittent ones and 12.4 (SD = 2.1) for the persistent patients in the ADHD group, and 12 (SD = 1.9) in the clinical control group. There were no significant age differences between the two groups (*p* = 0.86). Thirty-one (83.8%) boys and 7 (16.2%) girls were in the ADHD group, whereas 15 (68.2%) boys and 7 (31.8%) girls were in the clinical control group. Male to female ratio in the ADHD group was 4/1. The loss of the healthy subjects was due to an inability to located them (*N* = 8) or to family members’ refusal to participate (*N* = 8).

Of the 37 patients, 22 (59%) had experienced suspension and/or expulsion during the previous school year. In the ADHD group, only seven were treated with methylphenidate. Almost half of our sample, 47% (*N* = 14) had at least one grade-level repetition during their school progression and 22 patients had received warnings of possible problems/suspensions/expulsions school, in the previous year before the follow-up. One was arrested and was in a prison for teenagers for stealing and the use of psychoactive substances for the third time at the time of data collection.

### Persistence of symptoms

Twenty-eight out the 37 ADHD patients at follow-up showed symptom persistence (75%) and 9 (25%) showed full remission. Symptom persistence was as follows: 13 (46.4%) fulfilled all diagnostic criteria (syndromic persistence), 10 (35.7%) fulfilled sub-threshold criteria symptoms (symptomatic persistence), and 5 (17.8%) had functional impairments (functional persistence).

The descriptive data of the participants and the characteristics of the ADHD group regarding the persistence or absence of symptoms are shown in Table 1.

**Table 1:** **Demographic and clinical characteristics of the control and ADHD groups, expressed as means ± SD or percentage (%)**.

	Control	ADHD		ANOVA or chi-square	*p*-Values
		Remittent	Persistent	
	(*N* = 22)	(*N* = 9)	(*N* = 28)	
IQ	122.5 (17.7)	83.2 (50.8)	90.8 (28.7)	*F*(3, 59) = 2.26	0.45
Age (years)	12 (1.9)	13.1 (2.5)	12.4 (2.1)^a,^*	*F*(3, 59) = 1.44	0.01**
ABEP	19.7 (1.9)	19.3 (3.6)	15.9 (4.6)^b,^*	*F*(3, 59) = 0.19	0.001*
School	*N* (%)	*N* (%)	*N* (%)	χ^2^(2) = 0.47	0.01**
Particular	12 (54)	1 (14)	18 (64)		
Public	10 (46)	6 (86)	9 (36)		
Medication	*N* (%)	*N* (%)	*N* (%)	χ^2^(1) = 0.47	0.49
Use	–	1 (88)	6 (78)		
No	–	8 (12)	22 (22)		

*IQ, intellectual quotient; ABEP, socioeconomic classification; GAF, global assessment of functioning*.

*Data were analyzed by ANOVA and chi-square test*.

*p ≤ 0.001; **p ≤ 0.01; ^a^Persistent vs. Controls; ^b^Persistent vs. Remittent.

### Frequencies of comorbidities

In the sample, 33.1% (13/37) had none of the investigated comorbidities, 13.5% (5) had at least one comorbid disorder, and 51.3% (19) had 2 or more disorders. In the persistent ADHD group, only 18% (5/28) did not have comorbidities.

### Associations among comorbid disorders and persistence of symptoms

Persistent ADHD was significantly associated with significant rates of ODD 70% (*n* = 19), CD 70% (*n* = 19), and affective problems, such as dysthymia (DD) and major depressive episodes (MDD) 74% (*n* = 20) compared with controls. Remitted patients did not differ from controls (see Table 2).

**Table 2:** **Prevalence of comorbidity at follow-up between control and ADHD groups**.

	Persistent (*N* = 27)	Remittent (*N* = 9)	Controls (*N* = 19)	Test statistic	*p*-Values
	*N* (%)	*N* (%)	*N* (%)	
Affective	20 (74)^a^**^,b^**	0 (0)	4 (21)	χ^2^(2) = 21.07	<0.001
Anxiety	15 (55)	3 (33)	7 (37)	χ^2^(2) = 2.21	0.33
Somatic	8 (30)	5 (55)	3 (16)	χ^2^(2) = 4.68	0.09
Attentional	23 (85)^a^**^,b^**	3 (33)	2 (10)	Fischer’s	<0.001
Oppositional	19 (70)^a^*^,b^**	1 (11)	5 (26)	χ^2^(2) = 13.25	0.001
Conduct	19 (70)^a^**^,b^**	0 (0)	1 (5)	χ^2^(2) = 0.47	<0.001

*^a^=vs. Controls; ^b^=vs. Remittent*.

*Data were analyzed by chi-square test and Fisher’s exact test*.

*p ≤ 0.05; **p ≤ 0.01.

## Discussion

In this 4-year follow-up, symptomatic persistence was associated with high rates of comorbidities, a low number of patients in treatment for ADHD and school problems.

In the past, it was believed that ADHD symptoms persisted until mild-adolescence, but now there are signs of their often continuing throughout adolescence and into early adulthood (44). Corroborating these findings, our results showed that three quarters of the current sample presented with some type of ADHD symptom persistence 4 years after the first evaluation, and almost half met syndromic persistence criteria. This rate of persistence differs slightly from other follow-up studies. Biederman et al. (30) reported a 35% persistence rate for full ADHD diagnosis and 22% when partial remission cases were included, and Mick et al., (8) found 71% persistence rates. Persistence rates found in this study may reflect the difficulty in recognizing mental health problems in children and adolescents by pediatricians and psychiatrists, and the small number of professionals specializing in child psychiatry in Brazil (45). Across the country, we have few public and private services available to make appropriate diagnoses and afford treatment. In addition, there is great concern in university and government sectors over the possible over-treatment of children and adolescents with ADHD in our country. However, in fact, it is estimated that only 16.2–19.9% of individuals affected by Brazil received first-line treatment for the disorder in 2009–2010 (46).

In reality, the actual number in treatment is probably lower still because these stimulants can also be used for other purposes, though less frequently (46). Thus, a patient with ADHD can remain undiagnosed and untreated for many years, and this may result in increased persistence of symptoms and development of comorbidities. However, these results seem to agree with previous reports documenting that ADHD is a highly persistent disorder (13, 17, 19, 22, 25, 47, 48) and that patterns of persistence and remission can be predicted over the long term into young adult life.

The patients included at baseline had a diagnosis of ADHD without comorbidities. Four years later, the comorbidity rate was 75%. This rate was higher than those found in other studies: 46.4% (49), 46.6% (35), and 50% (13). Such differences could perhaps be explained by sample characteristics because in this study the patients were selected from a center specialized in ADHD treatment, a reference in the area, thus potentially composed of patients with more severe forms of the illness and more thus more likely to seek treatment.

In this work, persistent ADHD was associated with significant rates of ODD, CD, and affective problems compared with controls. This result is consistent with previous studies showing that comorbidities, particularly behavioral problems, are predictors of lifelong ADHD persistence (7, 8, 22, 30, 31, 50). Although the disruptive behavior disorders (ODD and CD) are considered phenomenologically different, comorbidity is very common. Disruptive behavioral disorders (DBD) and ADHD are both characterized by certain patterns of misbehavior among adolescents. Studies have shown that the severity of CD symptoms is associated with comorbid DBD and ADHD. Adolescents diagnosed with comorbid DBD and ADHD had an increased risk for anxiety disorders, depressive disorders, and substance abuse disorders. The comorbidity between DBD and ADHD should be considered in clinical practice because it could indicate more serious problematic behaviors than in the pure disorders alone (51, 52).

Regarding ADHD’s comorbidity with mood disorder, recently studies have reported depression in 14% of ADHD children vs. 1% of those without ADHD (53). A review of comorbid depression in community samples has reported that the rate of MDD in youths with ADHD is 5.5 times higher than in youths without ADHD, with rates ranging from 12 to 50% (54). Overlapping symptoms between bipolar disorder (BD) and ADHD make differential diagnosis a complex clinical task (55). It is noteworthy that up to 20% of children diagnosed with ADHD meet the criteria for BD and 50% of adolescents with BD meet the criteria for ADHD (56, 57). The rate in this 4-year follow-up study differed from others. Possibly the characteristics of our sample could explain the higher rates of comorbidity with affective disorders (poverty, poor access to treatment, low use of medication, and maybe chronic frustration and disappointment of living with untreated or poorly managed ADHD). ADHD’s comorbidity with mood disorders (MDD, DD, and BD) emphasizes the need for early detection and intervention to prevent, delay, or reduce its severity and associated impairments associated over time.

In relation to schooling, more than half the experimental group participants had been suspended or expelled in the latest data collection year. Our results confirm previous work showing that ADHD in childhood is associated with dropping out of school, significantly higher rates of repeated grades, tutoring, placement in special classes, reading disability, and fewer years of schooling attained. Thus, ADHD may increase the risk of adverse educational outcomes related to these symptoms in childhood and adolescence (10, 58, 59).

We observed that the number of children on medication for ADHD was very small (seven patients). Five of the seven patients were of the most favored social class and possibly had easier access to medical services and medication purchase. Despite substantial evidence supporting the efficacy of stimulant medication for children with (ADHD), adherence to stimulant treatment is often suboptimal (60). Many children discontinue the use of medication within the first year due to adverse effects or do not realize the effectiveness of treatment (61). Furthermore, the parent usually makes the healthcare decisions for children, and only some adolescents could make their own decisions. Among the factors that lead to lower adherence to treatment are the following: belief that the symptoms are not a disorder; distrust of the medical system; cost of medication; lack of providers in the community; and lack of insurance coverage (60, 62, 63). The data from this work confirm these findings and may be related to difficulties in access to treatment, medication purchase, and the lack of public policies in Brazil. Thus, there is necessity to improve the management of side effects, setting realistic treatment goals and constantly evaluating the success of therapy.

There are limitations to this study. The first is the relatively small sample size, occasioned by the strict inclusion criteria. A second limitation is the gender distribution with few girls, making it difficult to generalize the results. However, the predominance of boys in the sample is consistent with previous studies and in the general population. A third limitation is represented by the sample loss in the control group in the 4-year follow-up.

## Conclusion

Our results are relative to a population with potentially more severe ADHD (because they searched for treatment) and may not correspond with what happens with individuals with ADHD who do not seek treatment. The current analyses suggest that a small number of children with ADHD attain complete remission and the persistence of symptoms might be associated with more psychiatric comorbidities. This can indicate that the comorbid disorder is also persistent, and should encourage clinicians to carefully monitor comorbidities in children with ADHD and to be attentive to the residual manifestations of the symptoms of this disease over time. The results could suggest that a lack of effective pharmacological and psychosocial interventions and early diagnosis could aggravate the core symptoms of ADHD, leading to persistence of symptoms and the development of comorbidities, especially with ODD/CD. However, small number of children and adolescents with ADHD were treated effectively throughout the course of the disorder (64), and they tended to be so only for a short time. Thus, clinicians and pediatricians should be encouraged to carefully monitor children with ADHD and observe both manifestations of residual symptoms over time and comorbid-associated disorders. Furthermore, the improvement of the diagnosis of ADHD; frequent monitoring of cases already diagnosed; and the formulation of public health policies for these children and adolescents could have a substantial impact on the development of the disorder. In developing countries, such as Brazil, a better understanding of the long-term outcomes of children and adolescents with ADHD could help in the implementation of limited financial resources on programs and projects targeted to patients with poor outcome (cases of comorbidity, legal problems, abandonment school, etc.), (65).

## Conflict of Interest Statement

The authors declare that the research was conducted in the absence of any commercial or financial relationships that could be construed as a potential conflict of interest.
